# Lymphoblastoid Cell Lines as Models to Study Mitochondrial Function in Neurological Disorders

**DOI:** 10.3390/ijms22094536

**Published:** 2021-04-26

**Authors:** Sarah Jane Annesley, Paul Robert Fisher

**Affiliations:** Department of Physiology, Anatomy and Microbiology, La Trobe University, Bundoora, VIC 3086, Australia; P.Fisher@latrobe.edu.au

**Keywords:** lymphoblastoid cell lines, mitochondria, neurodegenerative disease, neurological disorder, cell models

## Abstract

Neurological disorders, including neurodegenerative diseases, are collectively a major cause of death and disability worldwide. Whilst the underlying disease mechanisms remain elusive, altered mitochondrial function has been clearly implicated and is a key area of study in these disorders. Studying mitochondrial function in these disorders is difficult due to the inaccessibility of brain tissue, which is the key tissue affected in these diseases. To overcome this issue, numerous cell models have been used, each providing unique benefits and limitations. Here, we focussed on the use of lymphoblastoid cell lines (LCLs) to study mitochondrial function in neurological disorders. LCLs have long been used as tools for genomic analyses, but here we described their use in functional studies specifically in regard to mitochondrial function. These models have enabled characterisation of the underlying mitochondrial defect, identification of altered signalling pathways and proteins, differences in mitochondrial function between subsets of particular disorders and identification of biomarkers of the disease. The examples provided here suggest that these cells will be useful for development of diagnostic tests (which in most cases do not exist), identification of drug targets and testing of pharmacological agents, and are a worthwhile model for studying mitochondrial function in neurological disorders.

## 1. Introduction

The mitochondria, tiny organelles present in all but a few human cell types, are best known as the energy powerhouses of the cell, responsible for producing more than 90% of the cell’s energy in the form of ATP. In addition to producing ATP, the mitochondria participate in other critical central metabolic pathways, such as apoptosis, generation of reactive oxygen species (ROS), division and proliferation, lipid biosynthesis and degradation as well as signalling networks, including calcium, hormone and immune signalling pathways. Given these essential and diverse roles, it is not surprising that mitochondrial dysregulation or dysfunction is linked to many diseases, including neurological and neurodegenerative disorders. Investigating mitochondrial function/dysfunction is key to understanding the pathogenesis of these diseases and has also been shown to be useful in both diagnosis and prognosis. 

Understanding of mitochondrial roles in disease has been greatly facilitated by the use of simple eukaryotic and animal models, including yeast, slime moulds, nematodes and flies [1,2,3,4,5,6,7,8]. These genetic models are based on gene mutations of genes associated with relatively rare familial neurodegenerative disorders and may not faithfully recapitulate what is happening in the more common sporadic cases of neurological disorders. This is supported by a number of therapeutic agents that only show benefit in familial disease models and not in sporadic disease cases [9]. 

In addition to these models, human cell lines, including primary cell lines, have provided invaluable insights into cellular disease mechanisms. Neurodegenerative and neurological disorders are characterised by death or dysfunction of neurons in the brain, but the underlying disease mechanism may be more systemic, also occurring in other cell types and tissues. This is supported by studies which show that peripheral tissues, such as blood, recapitulate the transcriptional changes that occur in the brain of patients with neurodegenerative disease [10,11,12,13]. The exact mechanism of the neuronal cell death is unknown, but in addition to the diverse collection of clinically described diseases arising from genetic forms of mitochondrial disease, mitochondrial dysfunction has been clearly implicated in many, including Alzheimer’s (AD) [14], Parkinson’s (PD) [15] and Huntington’s (HD) [16] disease as well as Fragile-X-associated Tremor and Ataxia syndrome (FXTAS) [17]. Mitochondrial dysfunction could be occurring systemically throughout the body in these disorders, but the effects would be more obvious in energy-demanding organs, such as in muscles and the brain. Neurons consume large amounts of energy and are postmitotic long-lived cells, which limits their regenerative capacity so that over time, mitochondrial dysfunction would lead to increased ROS production, increased mtDNA damage and worsening of mitochondrial function. This process of accumulated damage occurs naturally in ageing and is suggested to provide the link between neurodegenerative diseases and the biggest risk factor, ageing. Altered mitochondrial function in many neurodegenerative diseases is likely to be systemic, occurring in most tissues of the body. Indeed, the dysregulated expression of genes involved in metabolism and mitochondrial function has been identified in peripheral cells, such as blood and skin, of neurodegenerative disease patients [10,11]. 

If the mitochondrial dysfunction is systemic, then other, more accessible tissue may be used to characterise the dysfunction, use it as biomarkers of the disease and also to test the level or severity of the dysfunction in relation to clinical parameters. The most accessible tissue of the human body is blood. Compared to neurons, peripheral blood lymphocytes are short lived, rapidly turned over and, when activated, they become proliferative. These differences need to be taken into account when interpreting data from these cell types. In this review, the broad advantages and disadvantages of the various types of cell lines relative to lymphoblastoid cell lines (LCLs) will be briefly discussed. This will be followed by a discussion of specific neurological diseases where LCLs have been exploited to further our understanding of the underlying altered mitochondrial function present in all these diseases.

## 2. Types of Human Cellular Disease Models

There are many commercial cell lines available for use in the laboratory, and these are commonly obtained from cancerous tissue or are transformed with a tumour-inducing virus and have chromosomal abnormalities or mutations which enable them to continue proliferating in cell culture and are termed immortalised. The most commonly used and oldest cell line is the HeLa cell line, which was isolated from cervical cancer tissue from a patient called Henrietta Lacks in 1951 [18]. This cell line has contributed to many of the major advances in cancer biology and fundamental microbiology. It has also been used to expand our knowledge of neurodegenerative disease, such as characterising mitochondrial defects after expression of the mutant huntingtin (Huntington’s disease protein) [19] and also to study processes associated with neurodegeneration, such as autophagic clearance [20]. Whilst very useful and popular—a keyword search for HeLa cells in the last decade identifies more than 10,000 publications—this cell line has its limitations. It has been shown by several research groups that HeLa cell lines have diversified over the years and HeLa cells from different research groups are significantly different from one another, with differences in their genomes, mRNA expression levels and copy number variations (the number of repeats of a given gene) [21]. This raises the question of the reproducibility and generalisability, even to other HeLa cultures, of findings made in such a model. 

Another commonly used cell line is HEK293 and its derivatives, frequently used for its ease of culturing and transfection. Due to these attributes, HEK293 cells have been used to express mutant proteins associated with neurodegenerative disease, such as α-synuclein, LRRK2 and Tau in Parkinson’s and Alzheimer’s disease [22,23,24]. The HEK293 cell line was established from primary embryonic human kidney cells, transformed with sheared human adenovirus type 5 DNA [25]. The adenovirus DNA was integrated into human chromosome 19, and the cell line displays several chromosomal abnormalities, including extra copies of the X, 17 and 22 chromosomes, with the exact number of chromosomes differing between laboratory isolates [26,27]. 

Cell lines such as the catecholaminergic cell lines SH-SY5Y and PC12 are commonly used for studying aspects of neurological disorders, especially Parkinson’s disease, which affects the dopaminergic neurons. These cell lines have the machinery necessary to synthesise, release and store catecholamines, particularly dopamine, and can be differentiated into post-mitotic neuronal or neuron-like cells. SH–SY5Y cells were generated from a bone marrow biopsy of a metastatic neuroblastoma [28] and can be differentiated into a mature neuronal phenotype by several methods with the most common method involving exposure to retinoic acid [29]. SH–SY5Y cells have been used to understand PD disease processes and α-synuclein’s role in altered PD mechanisms (reviewed in [30]). The PC12 cell line was derived from a pheochromocytoma of the rat adrenal medulla. These cells are embryonic in origin but can differentiate into sympathetic ganglion neurons after culturing with nerve-growth factor [31]. They display many features of neuronal cells, they can synthesis and store dopamine, they express synapsin I protein, a marker of synaptic communication, and contain Toll-like receptors (TLR4), important in neuroinflammation [32]. These neuron-like differentiated cells have been used to model neurodegenerative diseases, including Alzheimer’s disease [33] and Amyotrophic Lateral Sclerosis [34]. These cell lines, whilst very useful, have some limitations. Like other cancerous cell lines, these cell lines also display a number of genetic aberrations [35]. Differences in growth, culturing and differentiation conditions can result in changes to the metabolome, proliferation and differentiation capacity [29,32] and these need to be taken into consideration when analysing results, especially across different laboratories, and could hinder use in high-throughput procedures. 

In contrast, Lund human mesencephalic (LUHMES) cells are not as susceptible to changes in culturing conditions and can also be induced to differentiate into neuronal cells. These cells are derived from human embryonic mesencephalon and were immortalised via introduction of a tetracycline-responsive v-myc gene [36]. The cells can differentiate into mature dopaminergic neuron-like cells by culturing them with tetracycline, cAMP and glial cell-derived neurotrophic factor (GDNF) [36]. Unlike the neuron-like cells discussed above, LUHME cells display electrical activities [37].

Whilst these commercial cell lines are extremely useful for studying gene function, the production of proteins and so on, they do not fully recapitulate what is happening in vivo and hence, primary cell lines and derivatives thereof may be more useful for modelling complex neurological human diseases. These models provide a tool for analysing cells taken directly from patients that retain genetic information, including mutations and epigenetic markers, which can then be compared to similar cell lines from healthy controls. Primary cell lines can be generated from a number of tissues, but the two most accessible human tissues are skin and blood. 

Skin tissue can be used to isolate and grow primary fibroblast cell lines, which can be cultured for up to *ca.* 50 cell doublings [38]. The replicative ability of fibroblasts in culture is affected by several factors, including the location of the body that the skin was isolated from [39], health [40], and in some reports, the age of the patient, with an inverse relationship between age and the proliferative ability of fibroblasts [41]. The number of culture passages they have passed through since isolation and the density of the culture from which they were drawn also affect the physiological state and replicative ability of fibroblasts [42]. Therefore, care must be taken when using fibroblast cells from patients to account for these differences.

By far the most accessible tissue of humans is blood, and samples can be taken on multiple occasions from the same participant. Peripheral Blood Mononuclear Cells (PBMCs) are routinely isolated from blood, and contain a mixture of lymphocytes (T cells, B cells, and NK cells) (70–90%), monocytes (10–20%), and dendritic cells (1–2%) [43]. The relative proportions of each cell type vary between individuals and are influenced by inflammation and disease-related processes. These cells do not survive outside the body and can only survive in laboratory conditions for about 5 days. The cells are not actively proliferating and are metabolically quiescent, so differences between patient and control groups may be obscured [44]. 

A limitation with all primary cell lines is that they do not grow indefinitely and, therefore, repeat sampling is often required, something which is not always possible and can result in participants being lost to the study. Once these cells are taken from the body, they begin a process of cellular ageing, culminating in senescence at rates that differ for different cell types, different individuals, and potentially even different participant groups (e.g., disease vs. control). Quite apart from, or even because of, the differences in their ex vivo and in vivo environments, ex vivo primary cell cultures and their in vivo counterparts cannot be considered equivalent. For example, from the few in vivo measurements that have been made, the rate of proliferation of fibroblasts in culture far exceeds their proliferation and turnover rates in vivo [38]. This implies corresponding differences in the in vivo and ex vivo metabolic and physiological states of the cells. So long as this is kept in mind, the study of primary cell lines can and has made many invaluable contributions to our understanding of mitochondria and neurodegenerative disorders. These include the use of fibroblast cell lines from PD patients to understand the function of a key PD-associated protein, PINK1 [45], and the use of PBMCs to detect altered gene expression of proteins to use as biomarkers of Alzheimer’s disease [46,47].

Multipotent stem cells, namely neuronal precursor cells (NPC) and oligodendrocyte precursor cells (OPCs), have been used to study neurodegenerative disorders, such as OPCs in ALS [48] and NPCs in Parkinson’s disease [49]. These precursor cells are extracted from the spinal cord, usually from embryos, and show some promise in therapies involving transplantation. With the development of induced pluripotent stem cells iPSCs by Takahashi and Yamanaka in 2006 [50], their use in modelling neurodegenerative disease has become widespread, and biobanked iPSCs exist for many neurodegenerative diseases. These cells have the benefit of being able to be made from patient cells. Initially, fibroblasts were used but now other cell types can be used, including the easily accessible PBMCs [51]. They can also be manipulated with gene manipulation technology, such as CRISPR, and therefore can model both sporadic and genetic forms of the diseases. During the creation of iPSCs and their subsequent induced differentiation into other cell types, genetic changes can occur, so care must be taken to screen them for major genetic rearrangements. However, smaller genetic changes are not so easily detected, so the possibility of their occurrence should be borne in mind [52]. Observing the same outcomes in multiple, independent iPSC lines can mitigate the risk of misleading results. Another important factor to keep in mind is that creating iPSCs requires the progenitor cells to be reprogrammed, and this is achieved by expression of several transcription factors which induce and maintain the pluripotent state [53]. The epigenetic modifications are removed and, as many neurodegenerative diseases seem to be caused by a mixture of genetics and environment, this may be a limitation of these cell lines for studying these disorders. The generation of iPSCs and differentiation into neuronal cell types and other cell types is still currently an expensive, laborious process which is out of the reach of many laboratories. However, the technologies associated with these cell lines are improving at a rapid rate and suggest that the cost and time involved will be reduced in the not-so-distant future. 

An alternative to these cell models is the transfection of PBMCs with Epstein–Barr virus (EBV) to generate continuously proliferating lymphoblastoid cell lines (LCLs). EBV selectively infects resting B cells, activates them and establishes a latent infection. The EBV genome has been reported to exist in these cells as distinct episomes, and the number of copies of the EBV genome was reported in a study of just under 1000 LCLs from the 1000 genomes project to be in the range of 20–29 [54]. Lymphoblastoid cell lines express several EBV proteins, and EBNA2 (EBV encoded nuclear antigenic protein) and LMP1 (latent infection membrane protein) have been shown to be essential in cell immortalisation, along with other latent phase EBV proteins. EBNA2 is a transcription factor that modulates expression of both viral and host genes, while LMP1 mimics a constitutively active CD40 to activate nuclear factor kB (NF-kB) [55]. Together, the EBV proteins promote B cell activation, cell proliferation and virus latency. 

As the virus exists in LCLs as an episome and expresses only a few viral proteins, there is minimal change to the host genome. Comparison of lymphoblastoid cell lines to their progenitor lymphocyte cells shows that the genomes are very similar with minimal difference between the two (99% similarity [56]). There is an increase in mtDNA copy numbers and also in mitochondrial mass, presumably in response to the cells transforming from resting cells into actively proliferating cells. The higher mitochondrial content makes the cells more oxidative than other cell types, such as fibroblasts, and as such, subtle differences in mitochondrial respiration are more likely to be detected in these cells. The somatic mutation rate is low (reported at 0.3% mutations per genome [57]) making them well suited to continuous culture in the laboratory setting. As the cells are transformed from resting cells into actively proliferating cells, many changes to gene expression occur to accommodate this, such as alterations in the levels of signalling proteins associated with activation, receptor activity and inflammation, cell cycle and metabolism. Whilst there are many changes in expression, the magnitude of the change is not large with one study reporting only 0.003% of transcripts with a magnitude change of 1.5-fold or higher [58]. Changes in methylation patterns are also observed with a general decrease in methylation levels in LCLs compared to B cells, as one might expect from cells that are actively proliferating and so need to be transcriptionally active. Despite these obvious differences, the methylation pattern and gene expression profiles of lymphocytes are largely maintained in LCLs and display many of the specific characteristics of primary B cells [58]. This is also true for many diseases, and clear differences in the transcriptomes and the epigenomes of patients and controls have been identified in LCLs for numerous diseases [59,60,61,62,63]. LCLs are increasingly being used as models of disease and given their high oxidative nature, are ideal systems for studying mitochondrial function. Many large facilities and biobanks now store large collections of LCLs from various disease and control groups in preference to other material, such as PBMCs (www.ecacc.org.uk; www.alspac.bris.ac.uk; http://cimr.umdnj.edu; www.lgcpromochem-atcc.com; www.rutgers.edu; www.coriell.org; access on 1 April 2021) [64]. LCLs were also used to provide the DNA for the 1000 genomes project. This review will now discuss some examples of how LCLs have been used to study mitochondrial function in several neurological disorders. 

## 3. Parkinson’s Disease (PD)

Parkinson’s disease is the second most common neurodegenerative disorder; it is characterised by death of neurons and accumulation of Lewy bodies in a specific part of the brain, the substantia nigra. The commonly held dogma has been that mitochondrial dysfunction is associated with Parkinson’s disease. Evidence for this came from autopsy studies of deceased PD brains [65], but post-mortem mitochondrial pathology could be a result rather than a primary pathological cause of the disease. In fact, almost all genes involved in familial PD do not encode mitochondrial proteins. Instead, they are involved in endocytosis and the trafficking of cellular membrane-bounded compartments, including but not limited to mitochondria [66]. Conversely, mutations in mitochondrial proteins do not typically produce PD as the disease outcome, but diverse other complex diseases [67]. Pharmacological studies with complex I inhibitors that result in elevated production of reactive O_2_ species (ROS) show that targeted mitochondrial damage can result in PD [68,69,70], but do not prove that this is the usual primary cause. It has been proposed that metabolically hyperactive parts of the brain (notably, the substantia nigra) are more vulnerable to such oxidative damage [71]. Notably, high mitochondrial respiratory activity also produces ROS damage at higher rates, suggesting the possibility that mitochondrial hyperactivity by functionally normal mitochondria could initiate mitochondrial damage early in the disease process [72]. 

Using LCLs derived from idiopathic PD patients and healthy controls, Annesley et al. [72] measured mitochondrial respiration directly in live cells. This revealed that, contrary to the commonly held belief that impaired mitochondrial respiration is an initiating cause of PD, LCLs from PD patients were, in fact, hyperactive with increased rates of mitochondrial respiration. All complexes in the ETC were functionally normal, while the mitochondrial mass and mtDNA copy number were unchanged. The elevated respiration was accompanied by an elevated ATP steady state and elevated ROS production [72]. Since that publication, the findings have been confirmed in LCLs from a larger cohort of idiopathic PD patients (unpublished data) and in other cellular PD models, including exposure of the neuroblastoma cell line SH–SY5Y to fibrillar alpha–synuclein [73] and in the simple eukaryotic model *Dictyostelium discoideum*, expressing genetically manipulated PD-associated genes, including α-synuclein [74] and DJ-1 [75]. In all cases, mitochondrial respiration was increased, not decreased. Another research laboratory has also identified increased basal oxygen consumption in fibroblasts from patients with LRRK2 mutations [76]. In support of elevated mitochondrial respiration, whole body respiration rates in iPD patients are known to be elevated [77]. The reasons for the observed increase in mitochondrial respiration in the iPD LCLs and the other PD models discussed above may be due to the fact that these cell types are proliferative and are rapidly renewed, unlike neurons. The authors propose that in early PD, perhaps before diagnosis becomes possible, increased mitochondrial respiration accompanied by elevated production of reactive O_2_ species (ROS) is present in peripheral tissues as well as the brain. Over time, this increased respiration in long-lived, terminally differentiated cells, such as neurons, could lead to an accumulation of ROS damage and mitochondrial dysfunction as observed in autopsied brains. In such a scenario, the mitochondrial pathology in PD brains is a result of initially undamaged, but hyperactive mitochondria [72]. In cells that are rapidly turned over, such as blood cells and fibroblasts, the damage does not have time to accumulate, so cells retain the elevated respiration state. As the increased respiration in idiopathic PD LCLs was not correlated with severity or disease duration, it is possible that the altered mitochondrial function is present prior to clinical diagnosis and as such, has the potential to be used as a predictive marker before large-scale neuronal loss has occurred and before presentation of clinical symptoms [72]. This hypothesis would need to be confirmed in other systems.

Familial PD, like other neurodegenerative disorders, accounts for a small percentage of cases. Of the genetic causes of PD, mutations in the LRRK2 gene are the most common [78]. In addition to familial PD, increased LRRK2 function is implicated in idiopathic PD and several clinical trials using LRRK2 inhibitors have begun. Detecting increased LRRK2 activity is difficult given the low levels of protein expressed, and measurements of substrate phosphorylation are used as indirect measures. LRRK2 is expressed abundantly in lymphocytes and in LCLs. Using LCLs from PD patients with LRRK2 mutations, Gonzalez-Hunt et al. [79] showed that these cells displayed an increase in mtDNA damage which could be reversed by treatment with RA334 a LRKK2 inhibitor. The authors of that study suggest that mtDNA damage levels in LCLs could be a sensitive cellular readout of altered LRRK2 kinase activity and could be used to measure LRRK2 kinase activity in clinical trials with LRRK2 kinase inhibitor-targeted approaches [79,80]. Together, these studies highlight the use of LCLs to identify biomarkers of PD.

One limitation of the LCL models for PD is that they have not been reported to exhibit the formation of Lewy bodies (LB)—characteristic intracellular inclusions in PD neurons that contain α-synuclein as one of their major constituents. However, this apparent lack of Lewy bodies is a feature of almost all PD models, both cellular and whole animal [81]. In fact, it seems increasingly clear that short, oligomeric species of α-synuclein are the cytotoxic form and not the large, insoluble aggregates in Lewy bodies [82,83]. In fact, Lewy bodies may have protective properties because they can sequester otherwise toxic oligomers. This being so, the absence of Lewy bodies does not prevent LCLs or other PD models from being valuable in the study of PD cytopathology.

## 4. Huntington’s Disease (HD)

Huntington’s disease (HD) is an autosomal neurodegenerative disease caused by a polyglutamine repeat expansion at the N terminus of the Huntington’s disease gene (*HTT*). The length of the polyglutamine expansion is negatively correlated with age of onset, and initiates a cascade of events leading, amongst other things, to impaired energy metabolism and mitochondrial function [84]. Although HD mainly affects the central nervous system, many peripheral tissues display abnormalities associated with the disease, suggesting that HD is a systemic disease [85]. Lymphoblasts express the Huntington protein, and lymphoblasts from HD patients display mitochondrial dysfunction. There are several lines of evidence that, at least in peripheral tissue, mitochondrial dysfunction occurs early in the disease process and may be centrally involved in disease pathogenesis [84,86,87]. 

In a small study by Mejia et al. [88], HD lymphoblasts were shown to have reduced mitochondrial respiration and reduced glycolytic function compared with healthy control lymphoblasts. This reduction did not correlate with the CAG repeat expansion length, but the sample size may have been too low to notice any such correlation (*n* = 8). Other studies have demonstrated that the mitochondrial membrane potential was reduced [87] which has since been shown in other HD models [87,89,90]. These authors also showed that HD lymphoblasts had a lower Ca^2+^ retention capacity than the controls, and when challenged with Ca^2+^, their mitochondria became depolarised at smaller calcium loads than the controls. They then expressed the Huntington protein with differing polyglutamine repeats in control lymphoblasts, and showed that the protein with an expanded repeat mimicked the diminished Ca^2+^ retention capacity seen in the HD lymphoblasts and in brain tissue from the HD mouse model [87]. Their results suggest that the Ca^2+^ defect may occur early in the disease process and may play a central role in the pathogenesis of the disease.

Using lymphoblast cell lines from HD and healthy controls, Sawa et al. [90] reported an increase in apoptotic cell death after stress induction and this was mediated through caspase-3 and caspase-8 and 9 activation [90,91,92]. The authors also reported an increase in mitochondrial depolarisation after stress.

Overall, lymphoblasts from HD patients do not display any morphological or growth differences from control lymphoblasts at rest, but when exposed to stress, mitochondrial differences and increased apoptosis are apparent. In a similar vein to what was proposed for PD lymphoblasts [72], Sawa et al. [90] also suggest that the reason that lymphoblasts appear normal is that lymphocytes are rapidly turned over. In the case of HD, this would mean that apoptotic lymphocytes are rapidly replaced, whereas in the brain the defective neurons are unable to be replaced [90].

## 5. Amyotrophic Lateral Sclerosis (ALS)

ALS is a neurological disease characterised by motor neuron loss in the brainstem, spinal cord and motor cortex [93]. Despite the clinical symptoms relating mainly to neuronal loss, ALS is considered a multi-systemic disorder, and many non-neural cell types, including peripheral cells, are suggested to be involved in triggering motor neuron loss [94]. ALS is a complex disorder and like other neurodegenerative diseases, no genetic cause is associated with the majority of cases (sporadic ALS, sALS); a small number of cases have a defined genetic component (familial ALS, fALS). Of the familial cases, mutations to several genes have been reported, including SOD1 (Super Oxide Dismutase 1), TARDBP (TAR DNA Binding Protein) mutation) and FUS (Fused in Sarcoma), with only the SOD1 mutations being specific to ALS [95]. Both sporadic and familial forms of the disease present with the same clinical manifestations, suggesting a similar disease pathogenesis, but studies in LCLs suggest that different pathological pathways exist in sALS and specific gene mutations in fALS. 

Investigation of LCLs from sporadic and familial ALS patients revealed that the different groups displayed different mitochondrial abnormalities. LCLs from sporadic patients displayed smaller mitochondria with unaffected respiratory function; LCLs with a SOD1 mutation were also smaller and rounder but also displayed an increase in mitochondrial respiration, a decrease in spare respiratory capacity and decreased glycolysis. Like the SOD1 mutation, LCLs from patients with a TARDBP mutation had increased mitochondrial respiration and a reduced spare respiratory capacity but had no change to their glycolytic flux. Finally, LCLs from patients with a FUS mutation displayed large mitochondria [94]. 

Another study by Guareschia et al. [96] using LCLs showed that in a subset of sALS patients, SOD1 was modified posttranslationally and was oxidised above baseline oxidation levels, and that this correlated with bulbar onset. Bulbar onset is the term used for ALS patients whose symptoms begin with speech and/or swallowing problems. This mimicked the familial form of ALS in which SOD1 is mutated, suggesting a common disease pathway for a subset of sALS patients and the SOD1 mutation fALS patients. 

Together, these results suggest that LCLs could be a useful model to identify and study how sALS and specific gene mutations affect particular disease pathways in ALS. This could be useful not only for increasing our knowledge of the underlying disease pathways but also in diagnostics and the development of therapeutics.

## 6. Neurodegenerative Diseases of the Optic Nerve

The optic nerve is part of the central nervous system (CNS) and its job is to transmit visual information obtained by the retina to the brain. The optic nerve is made up of glial cells and retinal ganglion cells (RGC), the latter of which are one of the most highly metabolic cell types. The RGC axons are unmyelinated and, therefore, require large amounts of energy or ATP to propagate action potentials. They have a high density of mitochondria and obtain their energy mainly from mitochondrial oxidative phosphorylation. These cells are particularly sensitive to mitochondrial dysfunction; external stressors and atrophy of RGCs due to mitochondrial dysfunction is associated with several diseases of the eye, particularly Primary Open-Angle Glaucoma (POAG), Leber’s Hereditary Optic Neuropathy (LHON) and autosomal dominant optic atrophy (ADOA). LHON and ADOA are primarily caused by mutations in mitochondrial genes; LHON is caused by mutations to complex I genes [97] and 60–70% of ADOA cases are caused by mutations in the dynamin-like GTPase protein Optic Atrophy 1 (OPA1) [98]. OPA1 is a fission–fusion protein involved in mitochondrial inner membrane remodelling. A small fraction, about 5%, of POAG has been attributed to single gene mutations in two genes: *myocilin* (*MYOC*) [99,100] and *optineurin* (*OPTN*) [100], and other genes have been identified as risk factors [101]. The mutated proteins in all these diseases are present in all cells, not just RGCs, and can be observed in peripheral tissue, such as blood cells. Why the mutations result in apoptosis only of RGCs is unknown, but it is postulated to be due to the high energy demand of these cells. Mitochondrial dysfunction is also implicated in POAG and the three diseases share phenotypic similarities.

Analysing primary patient tissue for these diseases is limited to post-mortem biopsies. There is a limited amount of tissue and a small number of samples available for research, so the modelling of these diseases in peripheral tissues has been extremely useful; the LCL model has been used extensively. 

Using LCLs, a specific Complex I defect was identified in all three diseases [102,103,104]. This defect was milder in POAG LCLs when compared to LHON LCLs and the authors speculate that this may reflect the slower rates of optic nerve loss in POAG patients when compared to the rapid optic nerve loss in LHON patients [104]. The LHON LCLs also displayed significantly higher NADH levels and decreased NAD^+^/NADH ratios compared to controls, suggesting that the higher mitochondrial defect in LHON cells compared to POAG cells is also accompanied by an altered redox state [104]. A subsequent study by Lopez Sanchez et al. [105], however, detected no difference in NAD^+^/NADH ratios in LHON LCLs compared to controls, so it is as yet unclear how redox potentials are changed.

As many as 220 different mutations to the *OPA1* gene have been identified in ADOA patients, and patients present with marked variation in their clinical phenotype with varying degrees of vision loss [103]. Differences in visual acuity are even observed amongst siblings harbouring the same OPA1 mutation. Using LCLs, Van Bergen et al. [103] showed that patients harbouring an OPA1 mutation and presenting with severe vision loss displayed reduced Complex I- and Complex II-driven ATP synthesis rates, whereas patients with the same mutation but with no vision loss had normal ATP synthesis rates. Furthermore, patients with the OPA1 mutation and normal vision had an elevation in Complex II and III activity and an increase in Complex IV protein, suggesting that in these people, the upregulation of the downstream complexes in the ETC (Electron Transport Chain) can compensate and bring ATP production rates to normal levels. Identification of genetic variants that enable this response may provide novel therapeutic insights into OXPHOS compensation for preventing vision loss in optic neuropathies. 

## 7. Fragile X-Associated Tremor Ataxia Syndrome (FXTAS) 

Fragile X-associated Tremor Ataxia Syndrome (FXTAS), first described in 2001, is a common neurodegenerative disorder affecting people, more commonly males, over the age of 50 [106]. FXTAS develops in people with an expanded CGG repeat in the 5′UTR of the *FMR1* gene. The human genome normally includes less than 40 CGG repeats in this region, but people with between 55 and 200 repeats are termed premutation carriers (PM) with around half of PM carriers developing FXTAS. Repeat numbers from 41 to 54 are referred to as Gray Zone alleles, and individuals carrying these have an elevated risk of developing a form of PD [107,108,109]. When there are more than 200 repeats, the *FMR1* gene is fully methylated, resulting in loss of the FMR1 protein and development of Fragile X retardation syndrome.

The expanded repeat found in PM individuals leads to increased transcription of the FMR1 mRNA; CGG expansion and FMR1 mRNA levels positively correlate. This correlation was observed in human lymphoblastoid cell lines of PM carriers as was an inverse correlation between FMRP protein levels and repeat length [110]. The mechanism behind the increased transcription of FMR1 mRNA in PM carriers was investigated in LCLs derived from PM carriers. The authors [111] identified an increase in acetylation of H3 and H4 histones at the FMR1 locus surrounding the CGG expansion, which positively correlated with FMR1 mRNA expression. They also confirmed this in another cell type—fibroblasts. Acetylation of histones is dynamic and reversible, and the authors showed that when they treated these cells with histone acetyltransferase (HAT) inhibitors, the amount of histone acetylation decreased at the FMR1 locus and reduced FMR1 mRNA expression to control levels. This finding opens up possible treatment options and identifies a previously unknown mechanism for how FMR1 mRNA is upregulated in PM carriers.

Using LCLs generated from PM carriers with or without FXTAS, [112] detected an elevation of mitochondrial respiration in PM carriers irrespective of FXTAS status and this correlated with white matter hyperintensities in the brain (WMH). WMH positively correlated with CGG expansion and mRNA FMR1 levels. No increase in mitochondrial mass was observed and overall, no functional difference in respiratory enzyme complexes was found, suggesting an upregulation of mitochondrial activity. This is reminiscent of what was observed in LCLs generated from PD patients [72]. Interestingly, one of the non-syndromic features of FXTAS patients is parkinsonism. The strong correlation with clinical measures suggests an importance in pathogenesis of the disease. A follow up paper by the same authors [113] went on to identify elevated AMPK activity levels in PM individuals but only in the non-syndromic FXTAS and non-affected PM carriers. PM carriers with FXTAS displayed normal levels of AMPK activity. The decrease in AMPK from non-FXTAS to FXTAS individuals correlated with symptoms and white matter lesions in the brain, but not with CGG expansion size or mRNA FMR1 levels. These studies identify a new pathological mechanism in FXTAS and in PM carriers, and opens up a new area for further research and therapeutic development.

## 8. Myalgic Encephalomyelitis/Chronic Fatigue Syndrome (ME/CFS)

ME/CFS is a complex disorder characterised by fatigue lasting greater than 6 months, and, in most classification systems, an overwhelming post-exertional malaise (PEM). The exertion itself can be relatively minor, could be either physical or cognitive, and the malaise can occur immediately following the exertion or up to 3 days later. PEM is defined as an exacerbation of the individual’s ME/CFS symptoms. Patients suffer from a range of symptoms which include severe fatigue, musculoskeletal pain, headaches, recurrent flu-like symptoms, gastrointestinal problems, sensory sensitivity, concentration/memory difficulties and unrefreshing sleep [114]. Mitochondrial dysfunction has been investigated in ME/CFS using blood cells, muscle tissue, urine and saliva and in most cases, a defect in mitochondrial function was suggested, but no precise mechanism was identified. Direct measures of respiration in non-proliferating, quiescent cells, such as PBMCs and muscle cells, revealed no clear, consistent differences between patients compared with healthy controls [115,116,117,118]. However, studies using LCLs generated from ME/CFS patients unveiled a clear and specific defect in the efficiency of the final complex in mitochondrial respiration, Complex V [44]. It seems likely that this defect was able to be observed due to the cell model used—LCLs. LCLs are actively proliferating and therefore, metabolism, including respiration, is occurring at an elevated rate, so the differences between patient and control groups can be identified [44]. The patient cells also had increased activity of a key stress sensing regulator called Target of Rapamycin Complex 1 (TORC1 or, in mammalian cells, mTORC1 [44]). TORC1 is able to activate transcription factors and separately upregulate the translation of many proteins, including mitochondrial proteins. All five mitochondrial respiratory complexes were indeed upregulated, as were enzymes involved in the TCA cycle, fatty acid uptake, β-oxidation and other pathways that serve as alternatives to glycolysis involved in provisioning the mitochondria with oxidizable substrates [59]. This elevation of expression may represent a cellular “attempt” to compensate for the inefficiency of Complex V. The upregulation of mitochondrial proteins was also reported in non-immortalised lymphocytes and saliva, supporting the utility of LCLs as a model system for this disease [60,118,119]. Using measures of mitochondrial function and TORC1 activity in LCLs in combination with a cell death rate assay in frozen lymphocytes, the authors showed that these parameters could be used to identify or diagnose ME/CFS patients from healthy controls with a nearly 100% specificity and sensitivity [120]. If further validated, this could be developed into the world’s first diagnostic test for ME/CFS.

## 9. Conclusions

Neurodegenerative and neurological disease are one of the major causes of death and disability worldwide, and their incidence is expected to grow over the coming years due to the ageing population. Altered mitochondrial function has been implicated in most of these diseases, but key information about how this altered function occurs, whether it is a cause or an effect, how it differs in different neurological conditions and how the dysfunction progresses is lacking. Studying these processes is difficult due to the inaccessibility of brain tissue, which is the key tissue affected in these diseases. Cellular models have been and will continue to be useful systems in which to study mitochondrial function in these disorders. Each cellular model comes with its own benefits and limitations. Here, we focussed on the use of LCLs to study mitochondrial function in neurological disorders. These cells can be generated easily and cheaply from patient and healthy control blood samples, allowing the more common sporadic cases of the diseases to be studied. They have long been used as tools for genomic analyses, but here, we described their use in functional studies, specifically in regard to mitochondrial function (summarised in Table 1). LCLs are proliferative, metabolically active and have a high mitochondrial content, unlike the PBMCs from which they are generated. This may magnify or unveil cryptic differences between the disease and control groups. These models have enabled characterisation of the underlying mitochondrial defect, identification of altered signalling pathways and proteins, differences in mitochondrial function between subsets of particular disorders and identification of biomarkers of the disease. The examples provided here suggest that these cells will be useful for the development of diagnostic tests (which in most cases do not exist), identification of drug targets and testing of pharmacological agents, and are a worthwhile model for studying mitochondrial function in neurological disorders.

## Figures and Tables

**Table 1:** Mitochondrial changes in LCL models of neurological disorders.

Disease	Genetic Mutation	Mitochondrial Changes	Ref.
Parkinson’s disease	idiopathic	Increased mitochondrial respiration, increased ATP steady state levels, Increased ROS, Increased AMPK activity	[72]
	LRRK2 (Leucine Rich Repeat Kinase 2) mutations	Increased mtDNA damage which could be reversed after treatment with LRRK2 inhibitor	[79,80]
Huntington’s disease	Patients with CAG expansion in Htt (Huntington) protein	Reduced mitochondrial respiration and reduced glycolytic function	[88]
		Reduced mitochondrial membrane potential, lower Ca^2+^ retention capacity, mitochondria depolarise after Ca^2+^ challenge at lower concentrations than controls	[87]
		Increase in apoptotic cell death and mitochondrial depolarisation after stress induction	[90]
	Htt protein with expanded repeat expressed in control LCLs	Reduced Ca^2+^ retention capacity	[87]
ALS (Amyotrophic Lateral Sclerosis)	Sporadic	Small mitochondria	[94]
	SOD1 (Super Oxide Dismutase 1) mutation	Small, round mitochondria, increased mitochondrial respiration, decreased spare respiratory capacity and decreased glycolysis	[94]
		SOD1 modified posttranslationally and oxidised above baseline oxidation levels which correlated with bulbar onset.	[96]
	TARDBP (TAR DNA Binding Protein) mutation	Increased mitochondrial respiration, reduced spare respiratory capacity	[94]
	FUS (Fused in Sarcoma) mutation	Large mitochondria	[94]
POAG (Primary Open-Angle Glaucoma)	Unknown	Complex I defect, decreased mitochondrial respiration and ATP production	[102]
LHON (Leber’s Hereditary Optic Neuropathy)	Complex-I G11778A	Complex I defect, higher NADH levels Decreased NAD^+^/NADH ratios	[104]
		Unaffected NAD^+^/NADH ratios	[105]
ADOA (Autosomal Dominant Optic Atrophy	OPA1 (Optic Atrophy 1) mutation	Complex I defect, decrease in ATP synthesis in patients presenting with severe vision loss. Increase in Complex II and II activity and Complex IV protein in LCLs from patients with no vision loss	[103]
FXTAS (Fragile X-associated tremor ataxia syndrome)	Expanded CGG repeat (55–200) in the 5′UTR of the FMR1 (Fragile X mental Retardation 1) gene	Increased mitochondrial respiration	[112]
ME/CFS (Myalgic Encephalomyelitis/Chronic Fatigue Syndrome)	N/A	Complex V inefficiency. Upregulation of mitochondrial proteins, enzymes involved in the TCA cycle, fatty acid uptake, β-oxidation and other pathways that serve as alternatives to glycolysis involved in provisioning the mitochondria with oxidizable substrates. Increased TORC1 activity	[44,59]
